# An interactive workshop plus locally adapted guidelines can improve General Practitioners asthma management and knowledge: A cluster randomised trial in the Australian setting

**DOI:** 10.1186/1471-2296-9-22

**Published:** 2008-04-20

**Authors:** Siaw-Teng Liaw, Nabil D Sulaiman, Christopher A Barton, Patty Chondros, Claire A Harris, Susan Sawyer, Shyamali C Dharmage

**Affiliations:** 1 Department of Rural Health, The University of Melbourne, Australia; 2 Department of General Practice, The University of Melbourne and Department of Family and Community Medicine and Behavioural Sciences, University of Sharjah, United Arab Emirates; 3 Discipline of General Practice, University of Adelaide, Australia; 4 Centre for Clinical Effectiveness, Monash Institute of Health Services Research, Australia; 5 Centre for Adolescent Health and Department of Respiratory Medicine, Royal Children's Hospital, Melbourne, Australia; 6 Centre for MEGA Epidemiology, School of Population Health, The University of Melbourne, Australia

## Abstract

**Background:**

A cluster randomised trial was conducted to determine the effectiveness of locally adapted practice guidelines and education about paediatric asthma management, delivered to general practitioners (GPs) in small group interactive workshops.

**Methods:**

Twenty-nine practices were randomly allocated to one of three study arms. Australian asthma management guidelines were adapted to accommodate characteristics of the local area. GPs in the intervention arm (Group 1, n = 18 GPs) participated in a small group based education program and were provided with the adapted guidelines. One control arm (Group 2, n = 18 GPs) received only the adapted guidelines, while the other control arm (Group 3, n = 15 GPs) received an unrelated education intervention. GPs' knowledge, attitudes and management of paediatric asthma was assessed.

**Results:**

Post intervention, intervention arm GPs were no more likely to provide a written asthma action plan, but were better able to assess the severity of asthma attack (Group 1vs Group 2 p = 0.05 and Group 1 vs Group 3 p = 0.01), better able to identify patients at high risk of severe attack (Group 1vs Group 3 p = 0.06), and tended to score higher on the asthma knowledge questionnaire (Group 1 vs Group 2 p = 0.06 and Group 1 vs Group 3 p = 0.2). Most intervention arm GPs felt more confident than control GPs to manage acute asthma attack and ongoing management of infrequent episodic asthma.

**Conclusion:**

Using interactive small group workshops to disseminate locally adapted guidelines was associated with improvement in GP's knowledge and confidence to manage asthma, but did not change GP's self-reported provision of written action plans.

## Background

The prevalence of asthma in Australia is one of the highest in the world [1,2]. Asthma accounts for about one fifth of the total disease burden in Australian children aged 0 – 14 years [3]. Self-management education together with effective drug therapy can reduce morbidity and mortality [4]. Australian General Practitioners (GPs) see 90% of Australians at least once annually [5] and are ideally placed to coordinate the care of patients with asthma; however, they are still under-diagnosing asthma and under-treating with inhaled steroids.

In an attempt to standardise asthma education and management, and reduce variability in patient care, clinical guidelines have been developed in Australia [6,7] and internationally. Despite this, recent studies in Australia indicate that recommendations are often not implemented in clinical practice [8], suggesting a need for more effective and innovative dissemination and implementation strategies [9]. The local adaptation of national guidelines has been proposed as a way of reversing this trend as it provides the benefit of local ownership, while maintaining scientific validity [10].

The International Drug Education randomised controlled trial [11] suggested that improvements in asthma treatment are possible with an educational program for providers based on self-learning in small peer groups. A recent systematic review concluded that interactive workshops could result in moderate changes in professional practice [12]. However, the few randomised controlled trials (RCTs) involving continuing medical education (CME) for asthma management alone, have had limited success in improving health outcomes for patients [13]. Generally, multifaceted strategies have been shown to be most effective [14].

This study brings together two key strategies for improving management of chronic disease: small group workshops plus locally-adapted clinical guidelines. The aim of this paper is to examine the effect of this intervention on General Practitioners (GPs) knowledge and management of paediatric asthma.

## Methods

### Study design

**Figure 1:**
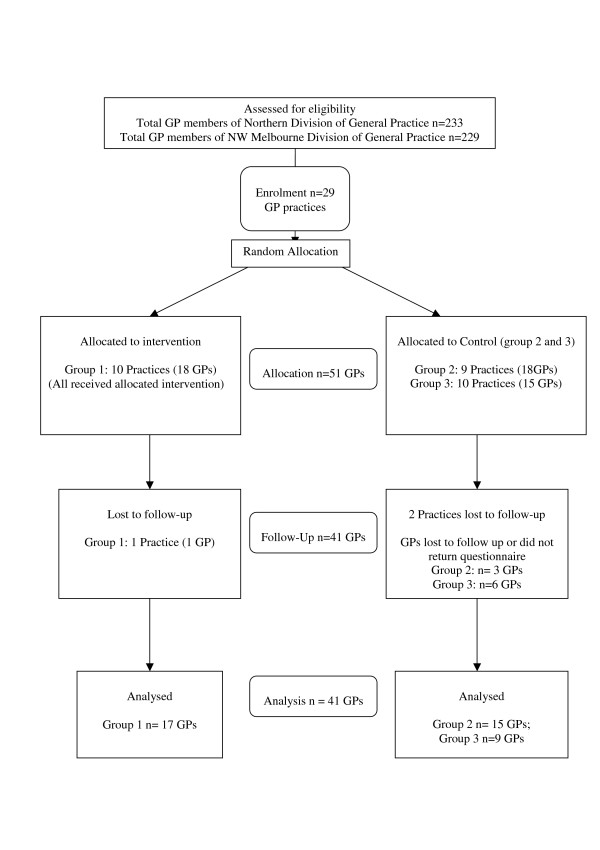
Randomisation of General Practices and General Practitioners.

A cluster randomised controlled trial (RCT) design was used, with practices randomised to one of three study arms (Figure 1). Group 1 participated in small group asthma education workshops and received locally adapted asthma management guidelines; Group 2 received the locally adapted asthma guidelines only and Group 3 received an alternative education program consisting of information about management of paediatric ear, nose and throat (ENT) problems. Group 3 did not receive any adapted asthma resource materials until the end of the trial. Approval for this study was given by the University of Melbourne Human Research Ethics Committee.

### Study Population and Recruitment

The trial took place between February and November 2001 in Melbourne, Australia. The Northern and North West Melbourne Divisions of General Practice and the Royal Australian College of General Practitioners (RACGP) promoted the trial via their newsletters, fax and e-mail circulars. GPs on the Divisions' membership lists were sent a personal invitation with a fax-back reply sheet. A telephone reminder from the study investigators followed this one-week later if the fax-back form had not been returned.

Patients with asthma aged between 2 and 14 years were recruited from each participating practice to assess the effect of the intervention on patient outcomes. Patient recruitment and outcomes were also assessed but are not reported in this paper. They will be published in an upcoming publication.

### Randomisation and blinding

The unit of randomisation was the general practice clinic. A table of random numbers was used to assign GP practices to study groups. It was not possible to blind GPs to which study group they had been assigned, however, patients were not informed by the investigators as to their GPs group allocation. Investigators were unable to be blinded to the group allocation of GPs, but were blind to the group allocation of patients.

### Description of the education program and guidelines

The paediatric asthma education program was developed using the resources available from the Royal Children's Hospital (RCH) Asthma Interest Group and the National Asthma Council [7], including the 6-step asthma management plan and adherence booklet. The education program included two workshops of approximately 3-hours duration that were held on a Saturday afternoon and Sunday morning. Presenters at the workshops included Paediatricians from the Melbourne Royal Children's Hospital, the Northern Hospital, and GPs. Twelve of eighteen eligible GPs attended the first program and a condensed program was provided to the remainder.

The paediatric asthma guidelines were adapted to the local context, a low socioeconomic area with a high proportion of culturally and linguistically diverse (CALD) groups, by an inter-Divisional group of GPs and the investigators. The guidelines were presented as flow-charts and dot points in three laminated A4 pages, printed on both sides. Approximately 12-hours of group discussion and several hours of individual review were required to achieve consensus on the guidelines.

### GP Outcomes and Instruments

GPs completed the survey instruments before the intervention and 6 months later. The primary outcome was GPs self reported use of Written Asthma Action Plans (WAAP) as part of the management of children with asthma. Secondary outcomes included knowledge of paediatric asthma that comprised 21 true/false statements about triggers of asthma in children and treatment of paediatric asthma and was adapted from the paediatric asthma knowledge questionnaire of Fitzclarence and Henry [15]. Additional questions were asked about knowledge of the severity of an acute asthma attack, which was made up of a checklist of 11 clinical features that GPs ranked as either a reliable indicator of severity, useful but less reliable, or unreliable or unrealistic feature. GPs were also asked to identify 7 indicators out of a list of 10 items that would make them concerned that a child was at 'high risk' of a severe attack. For each of these 3 outcomes, the number of correct responses were summed and transformed to percentage score. GPs were also asked to rate their confidence (very confident, not very confident, not confident at all) in managing a mild, moderate, or severe/critical asthma exacerbation in children, and their confidence in managing infrequent episodic, frequent episodic, and persistent asthma.

Other questions were asked about patterns of asthma, and diagnosis and management based on three case scenarios. The case scenarios presented paediatric patients presenting with three levels of asthma severity (infrequent episodic, frequent episodic and persistent). The education package, guidelines and questionnaire are available online [16].

### Sample size calculations

Sample size calculations for the trial were based on patient outcomes. The primary outcome was the proportion of patients with a written asthma action plan at 6 months post-intervention. We assumed an intra-cluster correlation (ICC) of 0.02 for the provision of asthma action plans in the sample size calculations, as at the time of the sample size calculations there was no published literature for the ICC estimate. To show a difference of 20% in the proportion of patients with written asthma action plans between intervention and control group, assuming that 50% of patients would have a written asthma plan in the control group, 80% power and significance level of 5% for a 2-sided test, we required 13 patients from each GP practice given that there were 10 GP practices in each arm. After allowing for non-response and loss to follow-up throughout the trial we required 17 patients per practice.

### Data Analysis

The data were analysed using Stata Statistical Software: Release 8.0 (Stata Corporation, College Station, Tx, USA). Demographic variables were summarised using frequencies and percentages for categorical data and median and inter-quartile range (IQR) for years in general practice. Fisher's Exact test was used to examine GPs level of confidence across the three study groups.

For the primary outcome of whether the GP had provided patients with a written asthma action plan (WAAP yes/no), logistic regression was used to calculate the odds ratio and respective 95% confidence interval (CI) and p-value comparing the intervention group to each control group. No adjustments were required for the clustering effect (arising from more than one GP participating from some Practices), as the estimated intra-cluster correlation for provision of WAAP was very close to zero. Linear regression was used to examine the difference in mean knowledge scores between the intervention group and each control group, adjusting for baseline outcome measure. In the regression analysis, the mean cluster score for each practice was used to allow for the clustering effect. Results were reported as difference in means with respective 95% confidence intervals (CI) and p-values.

## Results

GPs from 32 practices (n = 63 GPs) were initially enrolled, though three practices and 12 GPs dropped out of the study after patient recruitment. The flow of practices and GPs through the study is shown in Figure 1.

Demographic and other background information about the participating GPs is presented in Table 1 (see additional file 1). Practice factors and GP characteristics were generally well balanced across the three study groups, except for years in general practice where GPs in Group 3 tended to have more years in general practice than GPs allocated to Groups 1 and 2.

**Table 1:** Demographic and other background information about the participating practices and GPs at baseline

	**Group 1**		**Group 2**		**Group 3**	
	**n**	**(%)**	**n**	**(%)**	**n**	(%)
**Practice characteristics (N)**	10		9		10	

**Number of GPs participating from each practice (GP cluster size)**						
1	3	(30)	4	(44)	6	(60)
2	6	(60)	2	(22)	3	(30)
3–4	1	(10)	3	(33)	1	(10)
						
**Number of Solo GPs**	2	(20)	4	(44)	4	(40)

**GP Characteristics (N)**	18		18		15	

Gender (proportion of male GPs)	12	(67)	12	(67)	10	(67)
Graduated in Australia	10	(56)	10	(56)	10	(67)
						
Years in General Practice in Australia – Median (IQR)	11	(7, 16)	15.5	(10, 20)	19	(14, 25)
Years in General Practice in overseas- Median (IQR)	0.5	(0, 5)	1	(0, 5)	0	(0, 5)
						
Qualifications and Membership*						
Vocational registration	11	(61)	13	(72)	14	(93)
Fellowship/GP Training Program	15	(61)	7	(28)	4	(20)
Diploma/Master/PhD	2	(11)	6	(33)	5	(33)
Other	2	(11)	2	(11)	3	(20)
Sessions usually worked per week – Median (IQR)	9	(6, 10)	8	(6, 10)	8	(6, 10)

* Groups not mutually exclusive

There was no evidence supporting the hypothesis that GPs in the intervention group (education + guidelines) were more likely to report providing a written asthma action plan to children compared to GPs in the two control groups (Group 1 vs Group 2: OR = 1.15, 95% CI: 0.14 to 9.4, P = 0.89; and Group 1 vs Group 3: OR = 3.8, 95% CI 0.50 to 28.4, P = 0.20) (see additional file 2).

At baseline (pre-intervention) there were no differences between the groups in their asthma knowledge, assessment of asthma severity, or assessment of high-risk asthma (see additional file 3). Post-intervention, there was some evidence suggesting that GPs in the intervention group had increased their knowledge about asthma and were better able to assess the severity of asthma attack. Likewise, there was evidence suggesting that these GPs were better able to identify patients at high risk of severe attack compared to the control groups that received the alternate intervention (see additional file 3).

At baseline there were no differences between intervention and control groups in GPs self-reported confidence in managing acute asthma or routine management of asthma. The majority of GPs (> 87%) in all 3 groups felt 'confident' or 'very confident' treating and managing mild or moderate acute attacks and infrequent or frequent episodic asthma. However, 38% of GPs felt 'not very confident' or 'not confident at all' managing a severe/critical attack of asthma and 20% were 'not confident' about the ongoing management of persistent asthma. Post-intervention, a higher proportion of GPs in the intervention arm (Group 1) felt 'very confident' compared to control arm GPs for ongoing management of infrequent episodic asthma (p = 0.03), but there was no evidence to suggest that they were more confident managing acute attacks or ongoing management of frequent episodic or persistent asthma (see additional file 4).

No adverse events were encountered during the trial.

## Discussion

### Summary of main findings

This study found that simple, locally adapted best practice guidelines for paediatric asthma, disseminated during small group interactive workshops, can improve General Practitioners knowledge of asthma and confidence in triaging and managing patients with asthma, especially infrequent episodic asthma. However, certain elements of asthma management, in particular providing patients with a written asthma action plan, were not changed. This may be due to the relatively high self-reported use of written action plans (66%) in the intervention group at baseline and the characteristics of the GPs in the intervention group who tended to be relatively younger GPs who were more engaged with the Royal Australian College of GPs training program. The result may also be partly due to the nature of the intervention which focussed on education more so than behaviour change. Behaviour change may require more complex and multifaceted interventions that include practice organisation strategies to initiate, support and maintain change.

### Strengths and the limitations of this study

This paper focuses on the GP outcomes; however, the number of GPs in each arm is small. The small number of practices means our estimates for the provision of written asthma action plans, asthma knowledge and confidence scores are less precise than we would ideally like (as indicated by the wide confidence intervals), however, the effect sizes suggest that the intervention was associated with real improvement in knowledge and confidence in the intervention group when compared to the control group, and the proportion of GPs in the intervention group who reported using written asthma action plans increased (as hypothesised) although this change was not statistically greater than the change observed in the control groups. A larger sample size would be needed to determine if changes are a result of the intervention or due to chance.

The intervention was delivered at the practice level and main outcomes are self-reported, so we are unable to determine whether changes in knowledge and confidence translate into better clinical practice. The results may also be influenced by GP recall and positive responding (particularly in regard to provision of a written asthma action plan by the intervention group); however, no statistically significant differences were found in the self reported provision of a written asthma action plan. An alternative strategy would have been to audit practice medical records, but these can not be considered a gold standard since the practice records may be incomplete or inaccurate [17,18], and in any case, are not appropriate to assess GPs knowledge and confidence and so the additional expense of conducting an audit of records was not appropriate.

A common methodological issue in general practice and primary care trials is the Hawthorne effect [19]. This was addressed through the use of control practices that received an alternative intervention (ENT education in a similar guidelines and workshop format). In addition, the control group practices were motivated to collect data and discouraged from seeking other CME activities on asthma during the trial period. Despite this, 6 GPs in this control arm did not complete the trial.

Another potential problem for primary care based RCTs is that patients may not return to see the GP who participated in the educational intervention. To minimise the risk of this, one of our selection criteria was that at least 50% of GPs from any clinic must be enrolled in and complete the study. Hence recruitment occurred at clinic level with clinics selected and randomised, instead of individual GPs. This, however, also proved to be a barrier to participation, as some GPs who expressed interest in the project could not participate because partner GPs were either not interested or could not find the time. This was particularly true for the practices with three or more GPs.

Finally, GPs were self-selected and represented a small proportion of the 400-strong membership of the Northern and North West Melbourne Divisions of General Practice. This may limit the generalisability of this study.

### Implications for future research or clinical practice

Despite the development of a large number of clinical practice guidelines in the last 25 years, only a few have been rigorously evaluated in primary care settings with randomised controlled trials [20]. Similarly, reviews of the education literature show that while education can lead to improvements in confidence and competence of practitioners [21], there is a dearth of quality RCTs that have investigated the effectiveness of education interventions for asthma in primary care settings [13]. This is consistent with the education literature generally, where there is little high quality evidence on the impact of health care professional education on the quality of services or on patient health outcomes. Further analyses of data collected as part of our study are planned to determine if there are benefits to patients that flow on from the changes in knowledge and confidence reported by their GPs here.

## Conclusion

Evidence based clinical practice guidelines for the management of asthma have been available in Australia for more than a decade. However, there is evidence that care is still not in line with these guidelines. Our approach, which utilised small group education and dissemination of locally adapted guidelines, was associated with some benefits. GPs in this study were drawn from practices located in areas of high cultural diversity, and so this approach will be particularly useful to others seeking to improve GPs asthma knowledge and confidence in similar settings.

Understandably, this RCT which involved an educational intervention demonstrated improvements primarily in knowledge and confidence, but did not produce a statistically significant change in behaviour (i.e. the use of written asthma action plans), although a greater proportion of intervention group GPs reported utilising written asthma action plans 6 months after the intervention. It seems likely that further behaviour change may require an understanding of the readiness to change as well as ongoing support at the point of care.

## Competing interests

The author(s) declares that they have no competing interests.

## Authors' contributions

CH, SL, NS and SD contributed to conception and design of the study, interpretation of results, and critical review of the manuscript. CH was primarily responsible for the development and delivery of the intervention. CB was involved in the acquisition of data, some data analysis, interpretation of findings, and drafting the manuscript. SS was involved in the development of the intervention, interpretation of results, and critical review of the manuscript. PC provided advice on study design, conducted the statistical analysis and contributed to drafting the manuscript. All authors have read and provided comment on the final draft of the manuscript.

**Table 2:** GPs prescription of written asthma action plans and how they were used.

	**Group 1**		**Group 2**		**Group 3**		
	**n**	**(%)**	**n**	**(%)**	**n**	(%)	**P**
**Pre-intervention number of GPs**	*18*		*17*		*15*		
**Do you use Written Asthma Action Plans for children with asthma?**							
Yes	11	(61.1)	10	(58.8)	12	(80.0)	0.37
**If yes, how do you usually write an Asthma Action plan?**							
Standardised pre-printed form	4	(36.4)	3	(30.0)	5	(41.7)	
Computerised proforma	2	(18.2)	0	(0)	3	(25.0)	
Blank page	5	(45.5)	6	(60.0)	2	(16.7)	
Other	0	(0)	1	(10.0)	2	(16.7)	
							
**Post-intervention number of GPs**	*17*		*15*		*9*		
**Do you use Written Asthma Action Plans for children with asthma?**							
Yes	15	(88.2)	13	(86.7)	6	(66.7)	0.43
**If yes, how do you usually write an Asthma Action plan?**							
Standardised pre-printed form	7	(46.7)	6	(46.2)	2	(33.3)	
Computerised proforma	6	(40.0)	2	(15.4)	3	(50.0)	
Blank page	1	(6.7)	5	(38.5)	1	(16.7)	
Other	1	(6.7)	0	(0)	0	(0)	

Note one GP in Group 2 did not have baseline information (excluded from the denominator)

Group 1 = Asthma Education + guidelines

Group 2 = Guidelines only

Group 3 = Alternative (ear, nose and throat) education only

**Table 3:** Pre and Post intervention GP asthma knowledge in the intervention and control groups.

	**Pre intervention**		**Post Intervention**				
					
**Group**	N*	**Mean(SD)**	**N**	**Mean(SD)**	**Diff (95% CI)**		**P**
**Knowledge about asthma – Percentage correct responses out of 21 statements**							
Group 1	18	63.5(12.3)	16	80.8(13.1)	0		
Group 2	17	62.8(12.4)	15	71.1(11.6)	-10.7	(-21.9, 0.56)	0.06
Group 3	15	61.8(10.1)	9	70.2(14.5)	-7.6	(-19.6, 4.4)	0.20

**Assessment of severity of acute attack in children – Percentage of correct responses out of 11 statements**							
Group 1	18	30.9(11.9)	16	56.3(17.9)	0		
Group 2	17	35.6(13.1)	15	40.1(17.3)	-14.1	(-28.1, -0.01)	0.05
Group 3	14	33.1(13.3)	9	33.8(13.3)	-20.9	(-35.7, -6.2)	0.01

**Identification of a child with asthma that may be at high risk – Percentage of correct response out of 10 statements**							
Group 1	18	82.8(8.3)	17	89.4(9.0)	0		
Group 2	17	84.7(10.7)	15	89.3(9.6)	-0.02	(-6.9, 6.8)	1.00
Group 3	15	81.3(8.3)	9	82.2(8.3)	-7.2	(-14.5, 0.17)	0.06

Diff = Difference between means, adjusted for outcome at baseline; Reference group is Group 1

n = Number of GPs

SD = Standard deviation

95%CI = 95% Confidence Interval. Linear regression was fitted on the mean score for each practice.

Nb. One GP in Group 2 did not have baseline information (excluded from the total)

Group 1 = Asthma Education + guidelines

Group 2 = Guidelines only

Group 3 = Alternative (ear, nose and throat) education only

**Table 4:** GPs self reported confidence treating children with asthma

		**Post – intervention**						
		
		**Group 1** **(N = 17)**		**Group 2** **(N = 15)**		**Group 3** **(N = 9)**		P*
		
**Management of**		**n**	**(%)**	**n**	**(%)**	**n**	**(%)**	
Acute attack	Very confident	16	(94.1)	12	(80.0)	5	(55.6)	0.09
	Confident	1	(5.9)	3	(20.0)	3	(33.3)	
	Not very confident	0		0		1	(11.1)	
	Not confident at all	0		0		0		

Moderate acute attack	Very confident	13	(81.3)	11	(78.6)	4	(50.0)	0.30
	Confident	3	(18.8)	3	(21.4)	3	(37.5)	
	Not very confident	0		0		1	(12.5)	
	Not confident at all	0		0		0		

Severe/critical attack	Very confident	2	(11.8)	3	(20.0)	3	(33.3)	0.58
	Confident	11	(64.7)	7	(46.7)	3	(33.3)	
	Not very confident	4	(23.5)	5	(33.3)	3	(33.3)	
	Not confident at all	0		0		0		

**Ongoing management of**								

Infrequent episodic asthma	Very confident	14	(87.5)	9	(60.0)	3	(33.3)	0.03
	Confident	2	(12.5)	6	(40.0)	5	(55.6)	
	Not very confident	0		0		11	(11.1)	
	Not confident at all	0		0		0		

Frequent episodic asthma	Very confident	9	(33.3)	6	(40.0)	3	(33.3)	0.49
	Confident	8	(55.6)	9	(60.0)	5	(55.6)	
	Not very confident	0		0		1	(11.1)	
	Not confident at all	0		0		0		

Persistent asthma	Very confident	7	(43.8)	5	(33.3)	3	(33.3)	0.73
	Confident	9	(56.3)	8	(53.3)	5	(55.6)	
	Not very confident	0		2	(13.3)	1	(11.1)	
	Not confident at all	0		0		0		

## Pre-publication history

The pre-publication history for this paper can be accessed here:


